# Investigating oral nicotine pouch use among adults in Riyadh, Saudi Arabia: prevalence, awareness, susceptibility, and associated symptoms

**DOI:** 10.3389/fpubh.2025.1607656

**Published:** 2025-09-04

**Authors:** Hassan Alkharaan, Abdulaziz Alrubayyi, Majed Kariri, Mohammed Alasqah, Banna Alnufaiy, Hanadi G. Alzahrani, Khalid Gufran, Yasser Altkhais, Muteb Algharbi, Fahad Alarfaj

**Affiliations:** ^1^Department of Preventive Dental Sciences, College of Dentistry, Prince Sattam Bin Abdulaziz University, Al-Kharj, Saudi Arabia; ^2^College of Dentistry, Prince Sattam Bin Abdulaziz University, Al-Kharj, Saudi Arabia; ^3^North Riyadh Dental Complex, Ministry of Health, Riyadh, Saudi Arabia; ^4^Department of Preventive Dental Science, College of Dentistry, University of Ha’il, Ha’il, Saudi Arabia; ^5^Department of Public law, College of Law and Political Science, King Saud University, Riyadh, Saudi Arabia

**Keywords:** oral nicotine pouch, prevalence, awareness, perceptions, symptoms

## Abstract

**Background:**

Oral nicotine pouches (ONPs) have rapidly gained popularity as a novel nicotine delivery method. However, data on ONP use, awareness, and associated beliefs in Saudi Arabia remain limited. This study aimed to investigate these aspects among adults in Riyadh region.

**Methods:**

A cross-sectional survey was conducted in 2024 involving 831 Saudi adults (age ≥ 18 years). Participants completed a self-administered questionnaire assessing ONP awareness, prevalence, beliefs, and susceptibility as well as potential associated ONP use symptoms. Logistic and multinomial regression models were employed to analyze the associations between these variables.

**Results:**

Overall, 59.3% of participants reported awareness of ONPs, and 14.2% having used them. Males were more aware (ORadj = 1.97, *p* < 0.0001) and user (ORadj = 2.86, *p* = 0.03) of ONP than females. Similarly, younger adults (aged 18–29 and 30–39 years) demonstrated higher ONP awareness (ORadj = 4.67 and 4.88, respectively, both *p* < 0.0001) and use (ORadj = 6.91, *p* < 0.002 and 6.12, *p* < 0.003, respectively) compared to older adults (40–69 years). Additionally, 95.8% of ONP users were smokers, more likely to be cigarette (ORadj = 9.53, *p* < 0.0001) or e-cigarette (ORadj = 8.43, *p* < 0.0001) smokers. Approximately 60% of participants demonstrated susceptibility to ONP use, characterized by curiosity, limited knowledge of health risks, and potential willingness to use. Favorable beliefs about ONPs were more prevalent among users. Furthermore, a positive correlation was observed between the frequency of ONP use and the likelihood of experiencing associated symptom (*r* = 0.3, *p* = 0.0009), with abdominal symptoms being the most reported symptom.

**Conclusion:**

Given that 95.8% of ONP users in this study were smokers, these findings suggest a potential future role for ONPs as a harm reduction strategy within the context of smoking cessation in Saudi population in Riyadh. However, continuous surveillance and targeted public health interventions are crucial to mitigate the potential negative consequences associated with ONP use.

## 1 Introduction

For decades, the detrimental effects of tobacco use on global health have been well-documented. Tobacco consumption in any form remains a significant cause of morbidity and mortality, affecting millions of individuals worldwide annually (1, 2). A recent systematic analysis for the global burden of disease study revealed a death statistic of over 8 million people from a tobacco-related disease in 2019 alone (3). Highlighting the regional impact of this global issue, unfortunately, WHO statistical trends indicated a gradual increase in tobacco use among individuals aged 15 and older in Saudi Arabia, from 16.6% in 2000 to 17.4% in 2022. Notably, tobacco use in Saudi Arabia was more prevalent among males (28.4% in 2022) compared to females (2.1% in 2022) (2), However, the consumption of tobacco products showed a continued decline globally, with about 1 in 5 adults worldwide consuming tobacco compared to 1 in 3 in 2000 (1). Despite this positive global trend, a paradoxical rise in annual tobacco-related deaths is anticipated, because tobacco kills its users and people exposed to its emissions slowly (4).

While traditional cigarettes remain a public health concern, their dominance as the primary form of tobacco use appears to be waning, particularly among youth populations (5). In the recent decade, the emergence of an increasingly diverse array of nicotine-containing products has gaining popularity, including electronic cigarettes (E-cigs) (5), tobacco heating products (6), and oral nicotine pouches (ONPs) (7).

ONPs are pre-portioned pouches that share similarities with Snus, a traditional smokeless tobacco product. However, unlike Snus, ONPs are demonstrably tobacco-free, relying solely on nicotine, flavorings, sweeteners, and plant-based fibers for their composition (8). This unique characteristic translates to a convenient, discreet, and potentially palatable method of nicotine delivery, potentially appealing to a broader user base, particularly in environments where traditional tobacco use is restricted. Capitalizing on this innovation and the growing social stigma against conventional tobacco use, large tobacco companies have marketed ONPs as “tobacco-free” (7, 9), “tobacco leaf-free” (9, 10), or “all white” (10) alternatives. This marketing strategy positions ONPs within the emerging category of “modern oral” nicotine products, alongside established options like nicotine lozenges and gum (11).

ONPs entered the U. S. market in 2016, and as an emerging product, sales have witnessed a dramatic rise. Sales figures indicate a significant increase from 0.16 million units ($0.7 million) in 2016 to a staggering 46 million units ($200 million) within the first half of 2020 alone (12). With further growth reaching $808 million from January to March 2022 (13). This rapid growth in popularity highlights the evolving global market for ONPs, which is projected to reach $22.98 billion by 2030 (14). However, the non-targeted marketing of ONPs raises concerns, especially as they appear to be appealing to youth and young adult never-smokers. This raises concerns about the potential for initiation and increased use among these vulnerable populations.

The composition of nicotine within ONPs plays a crucial role in their potential for addiction. Unlike traditional cigarettes where nicotine exists primarily in its free-base form, ONPs can contain nicotine in both protonated and unprotonated forms depending on their pH (15). Protonated nicotine predominates at a pH below 6.0, while unprotonated (free-base) nicotine increasingly predominates as the pH rises above 6.0 (15). Compared with protonated nicotine, unprotonated nicotine readily passes through the oral mucosa epithelium, leading to faster and more extensive increases in blood nicotine levels (16). Consequently, some ONPs formulated with a higher proportion of unprotonated nicotine may pose a greater risk of nicotine dependence due to the enhanced bioavailability (16). This concern is particularly relevant for youth, who are a growing target demographic for ONPs (17, 18). Nicotine is a highly addictive substance, and its use during adolescence can negatively impact brain development, potentially priming the brain for addiction to other drugs (19).

While smokeless tobacco products, including ONPs, generally lack the typical tobacco-specific nitrosamines (TSNAs) found in combusted cigarettes, toxicological concerns regarding these products still exist (20). Unsmoked tobacco can still harbor TSNAs in various forms, including N-nitrosonornicotine (NNN), N-nitrosoanatabine (NAT), N-nitrosoanabasine (NAB), and 4-(methylnitrosamino)-1-(3-pyridyl)-1-butanone (NNK) (21). A recent study by Mallock et al. (22) detected the presence of TSNAs in over half (26 out of 44) of the analyzed nicotine pouch products. The highest measured concentrations, though relatively low, were 13 ng and 5.4 ng per pouch for NNN and NNK, respectively. Beyond nicotine and TSNAs, the presence of chromium and formaldehyde, both toxic substances, has also been documented in certain nicotine pouch products (7). The presence of any level of toxic elements raises concerns regarding potential health risks. Notably, NNN exposure has been associated with an elevated risk of esophageal tumors (20). However, the U. S. Food and Drug Administration has assessed the levels of these harmful constituents in ONPs as substantially lower than those found in cigarettes and other smokeless tobacco products, including moist snuff and snus. Consequently, ONPs are considered to pose a reduced risk of cancer and other serious health conditions compared to cigarettes and most smokeless tobacco products (23).

In summary, oral nicotine products typically comprise nicotine, flavorings, pH buffers, and filling agents, alongside trace amounts of toxic substances such as TSNAs, metals, and formaldehyde (7, 24). ONPs are introduced as a harm reduction strategy for adults who currently smoke cigarettes and/or use other smokeless tobacco products, with the potential benefit of reduced harm outweighing the risks associated with ONP use compared to cigarettes and/or use other smokeless tobacco products. Therefore, this survey study aims to assess awareness and susceptibility to ONP use among Saudi adults, while also investigating the side effects experienced by those who have used these products. The findings of this survey will provide valuable insights into the prevalence and patterns of ONP use within the Riyadh region of Saudi Arabia.

## 2 Materials and methods

### 2.1 Study population and procedure

A cross-sectional survey was conducted over a seven-month period, from April to November 2024, to investigate awareness, susceptibility, use patterns, and beliefs related to ONPs among Saudi adults residing in Riyadh, Saudi Arabia. The target population comprised individuals aged 18 years and older. A total of 831 participants were recruited using a multi-stage cluster sampling approach to ensure representativeness across Riyadh’s diverse districts by geographic location, age, and gender. Data were collected using a structured, web-based, close-ended questionnaire consisting of 19 items adapted from previously validated instruments (25–27). The questionnaire included two main sections: the first section captured demographic information, such as age, gender, and current use of other nicotine products; the second section assessed participants’ awareness of ONPs, susceptibility to use, usage patterns, beliefs, and symptoms experienced during ONP use.

To implement the sampling design, Riyadh was first divided into five main regions: North, South, East, West, and Central, based on the city’s 15 municipal administrative areas (Riyadh Municipality) (28). From each region, two districts were randomly selected, yielding ten clusters in total. Within each selected district, a two-stage recruitment strategy was employed. Trained data collectors approached potential participants in public spaces such as cafés, parks, and shopping malls and administered the questionnaire in person using digital tablets. Recruitment within these sites was guided by predetermined age and gender quotas to maintain balanced representation. To further expand reach, participants were also encouraged to share a secure link to the online survey with eligible contacts within their neighborhood social networks (e.g., classmates, community group members). This controlled recruitment process was closely monitored to prevent overrepresentation of any subgroup. All completed questionnaires were reviewed to remove duplicate entries, using phone numbers provided in the cosent form as unique identifiers, and to verify that respondents met the inclusion criteria (saudi adults aged 18 years or older residing in Riyadh city). To minimize sampling bias, the final sample from each district was adjusted to reflect the proportional population distribution according to the latest municipal census data (28). Informed consent was obtained from all participants, and all data were collected and stored securely. Ethical approval for the study was obtained from the Prince Sattam Bin Abdulaziz University Research Ethics Committee (approval No. SCBR-357/2024).

### 2.2 ONP awareness, susceptibility, beliefs and associated use symptoms

ONP awareness was assessed by presenting participants with a visual aid showing popular ONP brands, followed by the question, “Have you ever seen or heard of nicotine pouches before this study?” Participants answering “yes” were classified as aware; those selecting “no” or “not sure” were classified as unaware. Subsequently, all ONP non-users completed validated susceptibility measures (26), which included questions on interest in trying ONPs (“Are you interested in trying nicotine pouches?”), perceived information sufficiency regarding risks (“Do you feel you have sufficient information about the potential risks of nicotine pouches?”), and willingness to use if offered (“Would you consider using nicotine pouches if offered by a friend or readily available?”). Participants were categorized as non-susceptible if they indicated no interest, reported sufficient knowledge of health risks, and stated an unwillingness to use; all others were classified as susceptible (25, 26). Participants then rated their beliefs about ONPs on three constructs: perceived health risks, perceived value as a cessation aid, and perceived addiction potential. Responses were recorded on a Likert scale and categorized into “agree” (combining “strongly agree” and “agree”), “disagree” (combining “strongly disagree” and “disagree”), and “do not know.” Confirmed ONP users self-reported any previously identified use-related symptoms (24, 29, 30), including frequent coughing, oral irritation (e.g., white patches, gum irritation/recession, blisters), taste alteration, dry mouth, throat symptoms, or abdominal symptoms, with an option for “no symptoms” or “other symptoms” (A simplified table summarizing the described ONP survey items is provided in the Supplementary file).

### 2.3 Questionnaire development

As the questionnaire was adapted from previously validated English-language instruments (25, 27), a rigorous cross-cultural adaptation process was undertaken. An initial pilot study involving 50 participants, stratified by age and gender, was conducted through face-to-face interviews. This pilot phase aimed to ensure linguistic clarity, cultural appropriateness, and freedom from ambiguity of all Arabic-translated items. Questions pertaining to ONP beliefs and perceptions were initially structured into two-item scales for constructs such as perceived health risks, perceived value as a cessation aid, and perceived addiction potential (detailed in Supplementary file). These two-item scales demonstrated strong internal consistency in the pilot, with Pearson’s correlation coefficients (r) of 0.64, 0.70, and 0.86 for perceived health risks, cessation aid value, and addiction potential, respectively. Based on this robust reliability and to minimize respondent burden and potential redundancy, each belief construct was ultimately represented by a single, streamlined item in the final questionnaire. To assess the questionnaire’s temporal stability, we conducted a test–retest procedure with a subsample of 50 participants, administering the questionnaire twice, four weeks apart (84% response rate). Test–retest reliability for core belief and perception items yielded Pearson’s r = 0.73, with use-pattern questions excluded due to their expected variability over time.

### 2.4 Statistical analyses

Post-stratification weights were applied to align the sample with the demographic distribution of Saudi adults aged 18–69 years in Riyadh, estimated at 2.5 million of the 4.43 million Saudi residents, based on the latest census data (31). Three sets of weighted regression models were fitted: first, weighted logistic regression models examined factors associated with ONP awareness (aware vs. unaware) and ONP use status (user vs. non-user), using demographics and use of other nicotine-containing products as independent variables. Second, weighted multinomial logistic regression was conducted to explore associations between ONP-related beliefs and ONP susceptibility and use statuses. Covariates included in all models were selected *a priori* based on existing evidence of potential confounders for tobacco product awareness and use (25, 27). These covariates comprised demographic variables (age group and gender) and use of other nicotine-containing products (cigarettes, e-cigarettes and hookah). Multicollinearity was assessed before modeling by examining variance inflation factors for all independent variables which indicated an acceptable level of collinearity. Each belief was modeled separately with “disagree” as the reference category, adjusting for demographics and other tobacco product use. As all questionnaire items were mandatory for submission, missing data on key variables were negligible. Discrepant responses to two related questions (ONP use and associated symptoms) were identified, reviewed, and excluded (~3% of responses) to maintain data quality. All regression analyses were conducted using IBM SPSS Statistics Version 29. Correlation analyses and figure visualizations were performed using GraphPad Prism Version 10.

## 3 Results

### 3.1 Prevalence and covariates of ONP awareness and use

The prevalence of ONP awareness and use across genders, various age groups and other nicotine product use statuses are presented in Table 1. Furthermore, adjusted odds ratios (ORadj) are provided to quantify the associations between these variables and ONP awareness and use. Overall, 59.3% (*n* = 493) were aware of ONPs, 85.8% (*n* = 713) non-user ONPs, and 14.2% (*n* = 118) used ONPs. Results from the multivariable logistic regression models revealed that males were more aware (ORadj = 1.97, *p* < 0.0001) and user (ORadj = 2.86, *p* = 0.03) of ONP than females. Similarly, younger adults (aged 18–29 and 30–39 years) demonstrated higher ONP awareness (ORadj = 4.67 and 4.88, respectively, both *p* < 0.0001) and use (ORadj = 6.91, *p* < 0.002 and 6.12, *p* < 0.003, respectively) compared to older adults (40–69 years). Additionally, ONP users were more likely to be cigarette (ORadj = 9.53, *p* < 0.0001) or e-cigarette (ORadj = 8.43, *p* < 0.0001) smokers and less likely to be never-smoker (ORadj = 0.19, *p* = 0.006). Table 2 exclusively presents the sub-stratification of gender, age and nicotine product use among our identified ONPs users group. This analysis reveals that only 4.2% of ONP users were never-smokers, while the vast majority (95.8%) had a history of either previous or current smoking. Specifically, within this ONP user group, 73.7% were current or previous cigarette smokers, 56.8% were current or previous e-cigarette users, and 33.1% were current or previous hookah smokers. It’s important to note that these percentages are overlapping, as an individual ONP user could have used multiple other tobacco or nicotine products.

**Table 1:** Prevalence and correlates of ONP awareness and use.

Parameters	Overall %	Aware of ONP		ONP non-user		ONP ever used	
		(*n* = 493)		(*n* = 713)		(*n* = 118)	
		%	ORadj (95% CI)	%	ORadj (95% CI)	%	ORadj (95% CI)
Gender							
Male	63.70%	68.40%	1.97 (1.40, 2.78)****	79.20%	0.35 (0.13, 0.87)*	20.80%	2.86(1.14, 7.90)*
Female	36.30%	43.40%	Ref.	97.40%	Ref.	2.60%	Ref.
Age							
18–29 years	48.00%	62.90%	4.67 (3.04, 7.30)****	84.50%	0.14 (0.04, 0.44)**	15.50%	6.91 (2.25, 26.78)**
30–39 years	32.40%	71.70%	4.88 (3.03, 7.97)****	80.70%	0.16 (0.04, 0.49)**	19.30%	6.11 (2.04, 23.53)**
40–69 years	19.60%	30.10%	Ref.	97.50%	Ref.	2.50%	Ref.
Cigarettes							
Yes	19.60%	91.40%	7.12 (3.51, 15.29)****	46.60%	0.11 (0.05, 0.21)****	53.40%	9.53 (4.86, 20.14)****
No	79.40%	8.60%	Ref.	53.40%	Ref.	46.60%	Ref.
E-Cigarettes							
Yes	13.50%	80.40%	1.27 (0.62, 2.67)	40.20%	0.12 (0.05, 0.24)****	59.80%	8.43 (4.09, 18.48)****
No	86.50%	19.60%	Ref.	59.80%	Ref.	40.20%	Ref.
Hookah							
Yes	12.30%	79.40%	1.01 (0.51, 2.07)	61.80%	1.14 (0.59, 2.26)	38.20%	0.87 (0.44, 1.69)
No	87.70%	20.60%	Ref.	38.20%	Ref.	61.80%	Ref.
Never-smoker							
Yes	66.40%	48.00%	0.90 (0.44, 1.87)	99.10%	5.26 (1.67, 18.6)**	0.90%	0.19 (0.05, 0.59)**
No	33.60%	52.00%	Ref.	0.90%	Ref.	99.10%	Ref.

Boldface indicates statistical significance (*p < 0.05; **p < 0.01; ***p < 0.001; ****p < 0.0001). ONP, oral nicotine pouch; ORadj, adjusted odds ratio; CI, confidence interval. Each estimates were adjusted for all variables in the table.

**Table 2:** ONP ever used descriptive statistics.

Parameters	ONP ever used
	(*n* = 118)
	%^#^
**Gender**	93.80%
Male	6.80%
Female	
**Age**	52.50%
18–29 years	44.10%
30–39 years	3.40%
40–69 years	
**Nicotine product use (ever)**	95.80%
Cigarettes	73.70%
E-Cigarettes	56.80%
Hookah	33.10%
**Never-smoker**	4.20%

Descriptive statistics for ONP ever used group. # = the column for each parameter adds up to 100%.

### 3.2 Susceptibility and perceptions for ONPs use

Table 3 shows the prevalence of NP beliefs and their associations with ONPs susceptibility and use statuses. Approximately 60% (*n* = 503) of participants demonstrated susceptibility to ONP use, characterized by curiosity, limited knowledge of health risks, and potential willingness to use (25–27). Overall, over half of participants (54.6%) perceived ONPs as posing a health risk, while 25.4% believed they could aid in smoking cessation. However, most participants (70.2%) disagreed to the addictive potential of ONPs, and 84.5% acknowledged a lack of sufficient information regarding their dangers. Furthermore, 62.2% expressed a desire for additional information about nicotine pouches. Multivariate multinomial regression analysis revealed that ONP users expressed more positive perceptions regarding ONP health risks and smoking cessation benefits compared to other groups when disagreeing with a belief was the reference. Notably, agreement odds of ONPs health risks were significantly lower among ONPs users (ORadj = 0.13, *p* < 0.0001), and higher among the non-susceptible non-user group (ORadj = 14.56, *p* = 0.0005), while the susceptible non-user group exhibited an insignificant association with agreement (ORadj = 1.07, *p* = 0.83). Additionally, agreement odds about ONPs smoking cessation benefits were found to be higher among ONPs users (ORadj = 7.84, *p* < 0.0001), and lower among the non-susceptible non-user group (ORadj = 0.09, *p* < 0.0001) and the susceptible non-user group (ORadj = 0.56, *p* = 0.02). Furthermore, the susceptible non-user group demonstrated significantly lack of sufficient information about the dangers of ONPs (ORadj = 0.24, *p* < 0.0001).

**Table 3:** Comparative ONP-beliefs to susceptibility and use.

Beliefs	Overall %	Non-susceptible non-user		Susceptible non-user		ONP ever used	
		(*n* = 210)		*(n* = 503)		(*n* = 118)	
		Agree%^#^	ORadj (95% CI)	Agree%^#^	ORadj (95% CI)	Agree%^#^	ORadj (95% CI)
ONP pose a health risk							
Agree	54.60%	82%	14.56 (4.04, 93.90)***	54.30%	1.07 (0.56, 2.03)	25.50%	0.13 (0.06, 0.29)****
Not sure	38.40%	16.80%	2.67 (0.71, 17.73)	40.00%	1.42 (0.76, 2.68)	55.90%	0.42 (0.19, 0.89)*
Disagree	6.98%	1.20%	Ref.	5.70%	Ref.	18.60%	Ref.
ONP lead to addiction							
Agree	23.80%	29.10%	2.40 (1.46, 3.98)***	21.70%	0.69 (0.48, 0.99)*	25.40%	0.64 (0.37, 1.09)
Not sure	5.90%	0.60%	0.08 (0.01, 0.40)*	9.30%	12.07 (3.47, 76.98)***	0.80%	0.08 (0.01, 0.60)*
Disagree	70.20%	70.30%	Ref.	69.00%	Ref.	73.70%	Ref.
ONP help to quit smoking							
Agree	25.40%	5.20%	0.09 (0.04, 0.21)****	18.50%	0.56 (0.34, 0.90)*	72.00%	7.84(3.62, 16.84)****
Not sure	58.50%	65.70%	0.58 (0.36, 0.93)*	67.00%	1.77 (1.17, 2.69)**	20.30%	0.69 (0.33, 1.59)
Disagree	16.10%	29.10%	Ref.	14.50%	Ref.	7.60%	Ref.
Have you received sufficient information about the potential risks of ONP?							
Yes	15.40%	35.50%	5.06 (3.09, 8.34)****	8.90%	0.24 (0.15, 0.37)****	16.90%	1.55 (0.82, 2.86)
No	84.50%	64.50%	Ref.	90.90%	Ref.	83.10%	Ref.
Would you like to get more information ONP?							
Yes	62.20%	43.60%	0.29 (0.19, 0.44)****	62.80%	0.97 (0.71, 1.33)	84.10%	4.59 (2.79, 7.76)****
No	37.80%	56.40%	Ref.	37.20%	Ref.	15.90%	Ref.

Boldface indicates statistical significance (*p < 0.05; **p < 0.01; ***p < 0.001; ****p < 0.0001). ONP = oral nicotine pouch, ORadj = adjusted odds ratio, CI = Confidence Interval, # = the column for each belief adds up to 100%. Each belief was modeled separately and adjusted for susceptibility and use status.

### 3.3 ONPs associated use symptoms

**Figure 1:**
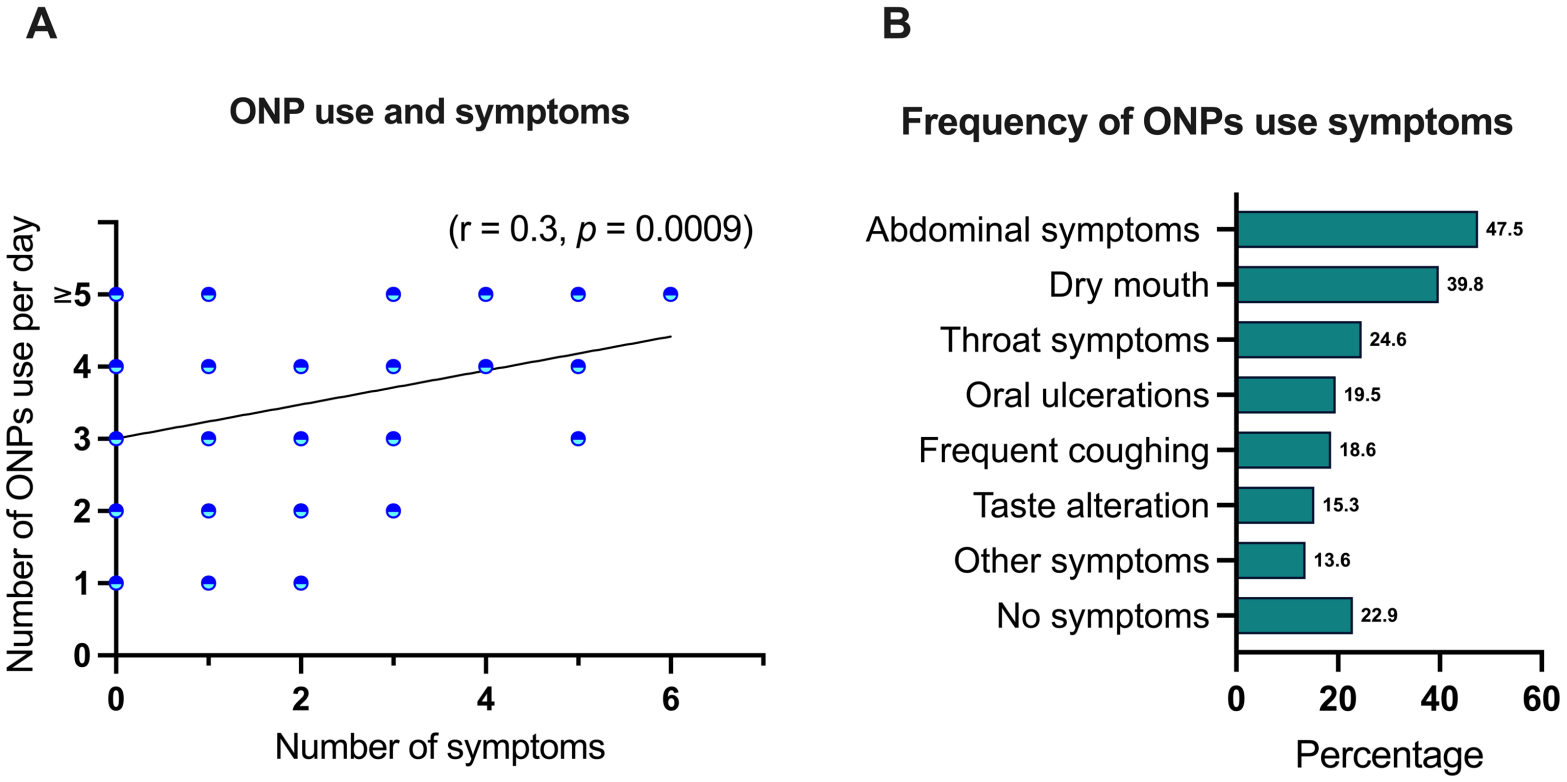
Correlation between ONP use frequency and the likelihood of experiencing associated symptoms were performed using Spearman correlation analysis **(A)**. Percentage of participants reported symptoms **(B)**.

A positive correlation was observed between the frequency of ONPs use and the likelihood of experiencing associated symptoms (r = 0.3, *p* = 0.0009) (Figure 1A). Among reported symptoms, gastrointestinal disturbances were most prevalent, with 47.5% of participants experiencing abdominal symptoms. Conversely, 22.9% of participants reported no discernible symptoms associated with ONP use (Figure 1B).

## 4 Discussion

This study represents the first investigation in Saudi Arabia to assess the prevalence of ONPs use, awareness, susceptibility, and associated symptoms among adult Saudis. The overall awareness rate of ONPs was found to be 59.3%, while 14.2% of participants reported ever using ONPs. These findings align with global trends, particularly in the United States, where awareness and use have significantly increased over the past few years among US adults who smoke. In 2020, awareness and ever-use rates in the U. S. were 19.5 and 3.0%, respectively (32), increasing to 29.2 and 5.6% between January and February 2021 (33) and further to 46.6 and 19.4% in 2021 (27) These relatively high rates of awareness observed in Saudi Arabia may be attributed to the active marketing of recently introduced local ONP products company that emphasizes ONPs as a potential aid for smoking cessation (34).

It is noteworthy in our study that 95.8% of ONP users were cigarettes, e-cigarettes, or hookah smokers, with significantly higher odds of ever using cigarettes (ORadj = 9.53) and e-cigarettes (ORadj = 8.43). This may indicate a potential public health impact in future for ONPs, as harm reduction strategy within the context of smoking cessation, in Saudi Arabia. However, continuous surveillance and targeted public health interventions are crucial to mitigate the potential negative consequences of ONP use. Notably, Keller-Hamilton and colleagues (35), reported that ONPs may not effectively facilitate tobacco cessation due to their potential for craving relief and their higher plasma nicotine delivery compared to traditional cigarettes, which could increase the risk of misuse.

Given these findings, the need for regulation of ONPs in Saudi Arabia becomes apparent. While ONPs are under the jurisdiction of the Saudi Food and Drug Authority (SFDA), they currently remain unregulated and are permitted advertising, unlike traditional cigarettes and other nicotine-delivery products (36). The impact of absence of regulation, especially in advertising, is evident in our study that while 59.3% of participants were aware of ONPs product, yet 84.5% reported a lack of sufficient knowledge of ONP potential dangers and 62.2% expressed a desire for more scientific information about the potential harms of ONPs. This knowledge gap may be attributed to the prevalence of promotional materials that present ONPs as a safe alternative to smoking.

We assessed in our study the comparative ONP-beliefs to susceptibility and use. Our findings indicate that between the 54.6% of participants who believed that ONP pose a health risks, only 6.6% of them (not shown in the table) were ONP ever users (ORadj = 0.13). Conversely, among the 25.4% of participants who believed ONPs aid in smoking cessation, 40.3% were ONP ever user (ORadj = 7.84). These results suggest that holding favorable beliefs about ONPs is associated with ONP use. Our findings align with previous study by Morean and colleagues which reported that young adults susceptible to or using ONPs were significantly more likely to hold favorable perceptions of ONPs compared to smokeless tobacco (ORadj = 2.54 and 2.18, respectively) (25). And to other studies that have reported a favorable ONP beliefs association to susceptibility to ONP use and awareness (27, 37). However, while our study suggests an association between specific beliefs to ONP use and susceptibility, it’s important to note that the susceptible non-user group held comparable beliefs agreement regarding ONP health risks and uncertainty about ONP potential addiction and smoking cessation aid (ORadj = 12.07 and 1.77; respectively). This warrants further longitudinal studies to explore this relationship and to confirm the long-term implications of these beliefs.

Several studies have investigated the potential side effects of ONPs highlighting some local and systemic health concerns. While no serious adverse effects have been reported, local oral irritations such as mucosal redness, ulcerations, gingival blisters, dry mouth, and sore throat have been observed, particularly with frequent or long-term use (38–40). Experimental animal and *in vitro* studies further suggest that nicotine may contribute to gingivitis (41) periodontal disease (42) and bone destruction (43), indicating that ONPs could promote periodontal inflammation. However, when used as a harm reduction strategy, self-reported outcomes among individuals who switched from traditional snus to novel ONPs with protective barriers showed reductions in mucosal lesions and gingival irritation (29). Similarly, a recent randomized controlled trial found that switching from smoking to ONPs significantly decreased signs of gingival inflammation and bleeding, implying a lower risk to oral health relative to cigarette smoking (44). Our study assessed the frequency of self-reported symptoms associated with ONP use and found a positive correlation between usage frequency and symptom prevalence (*r* = 0.3, *p* = 0.0009). Abdominal disturbances were the most common symptom, reported by 47.5% of participants, followed by dry mouth (39.8%), throat symptoms (24.6%), oral ulceration (19.5%), frequent coughing (18.6%), taste alteration and (15.3%). However, 22.9% of participants reported no discernible symptoms. Moreover, while systemic concerns have been raised, with a study of nicotine pharmacokinetic side effects of ONP use that reported cardiovascular effects of increased heart rate and elevated arterial stiffness indicating potential risks of increased arterial hypertension, atherosclerosis, and myocardial infarction, particularly in individuals with pre-existing cardiovascular conditions (24). switching from cigarettes to ONPs has been shown to substantially reduce exposure to harmful toxins in other a randomized controlled clinical study (45). Taken together, these evidence suggest that although ONPs are not entirely risk-free, they may serve as a less harmful alternative for individuals seeking to reduce or replace traditional tobacco use, underscoring the importance of further long-term research to fully evaluate their health impacts.

The ongoing evolution of novel ONP technology aims to mitigate specific risks and enhance user experience. Innovations like impermeable barrier technology (e.g., Stingfree PROTEX®) seek to reduce local oral irritation, with preliminary studies, including self-reported oral health outcomes following a switch from traditional snus or other pouches, suggest a reduction in mucosal irritation and improvement in gingival conditions with the use of such barrier-protected pouches (29, 46). Concurrently, optimized nicotine release and pH-optimized formulations (e.g., KLAR’s SERATEK) are being developed for a faster and potentially more efficient nicotine delivery with lower dose (47, 48). However, it is crucial to emphasize that no nicotine product is entirely risk-free, and extensive independent, long-term research remains essential to comprehensively validate claims, assess full toxicological impact, and ensure these innovations genuinely contribute to public health by supporting transitions from combustible tobacco rather than promoting novel nicotine use.

While this study offers valuable insights into awareness, use patterns, and beliefs regarding ONPs within Riyadh, its findings must be interpreted in light of several limitations. Given the novelty of ONPs and the emerging nature of related research, the study’s relatively small sample size—restricted to Riyadh—limits the broader generalizability of the results, although it is relevant to note that Riyadh alone represents 26.6% of Saudi Arabia’s population (31). Second, the unfamiliarity of ONPs to participants may have influenced their understanding, despite the inclusion of visual aids and “Not Sure” response options; thus, the true extent of public knowledge warrants further exploration. Third, the reliance on self-reported data inherently introduces potential biases, including recall and social desirability, which could affect the accuracy of reported outcomes. While our questionnaire provided detailed examples to enhance response consistency and underwent rigorous adaptation, pilot testing, and test–retest reliability analysis, residual measurement error cannot be entirely ruled out for subjective reports, such as oral irritation. Fourth, the absence of granular detail on participants’ comprehensive tobacco and nicotine use histories, specifically, data on frequency, duration, and precise current or former use, limits a more nuanced analysis of their overall tobacco use trajectories and the interplay with ONP uptake. Finally, the study did not account for participants’ underlying medical conditions, which may confound the interpretation of self-reported ONP-related symptoms, underscoring the need for future studies to integrate such contextual factors for more robust conclusions.

## 5 Conclusion

This study represents the first investigation into the awareness, susceptibility, and use of ONPs among Saudi adults in the Riyadh region, along with an assessment of associated side effects. While 59.3% of participants were aware of ONPs and 14.2% reported using them, it is notable that 95.8% of ONP users were current smokers, suggesting a potential positive role for ONPs as a harm reduction strategy within the context of smoking cessation in the Saudi population in Riyadh. Our findings also suggest a correlation between positive beliefs about ONPs and their use, while non-susceptible non-users hold more negative views. Moreover, While this study has identified potential side effects associated with ONP use, further research is needed to comprehensively assess both short-term and long-term health impacts. Given the potential risks associated with nicotine addiction and the limited long-term data on ONP safety, ongoing public health surveillance and targeted interventions are crucial to address the potential challenges posed by ONP use in Saudi Arabia.

## Data Availability

The original contributions presented in the study are included in the article/Supplementary material, further inquiries can be directed to the corresponding author/s.

## References

[1] World Health Organization. (2024). Tobacco. Available online at: https://www.who.int/news-room/fact-sheets/detail/tobacco (Accessed June 9, 2024).

[2] World Health Organization. WHO global report on trends in prevalence of tobacco use. 3rd ed. Geneva: World Health Organization (2024). 26 p. Available at: https://iris.who.int/bitstream/handle/10665/375711/9789240088283-eng.pdf?sequence=1

[3] GBD 2019 Risk Factors Collaborators. Global burden of 87 risk factors in 204 countries and territories, 1990-2019: a systematic analysis for the global burden of disease study 2019. Lancet. (2020) 396:1223–49. Available at: https://www.thelancet.com/journals/lancet/article/PIIS0140-6736(20)30752-2/fulltext 33069327 10.1016/S0140-6736(20)30752-2 PMC7566194

[4] Bilano V Gilmour S Moffiet T D’Espaignet ET Stevens GA Commar A. Global trends and projections for tobacco use, 1990-2025: an analysis of smoking indicators from the WHO comprehensive information systems for tobacco control. Lancet. (2015) 385:966–76. Available at: https://www.thelancet.com/journals/lancet/article/PIIS0140-6736(15)60264-1/fulltext 25784347 10.1016/S0140-6736(15)60264-1

[5] Ramôa CP Eissenberg T Sahingur SE. Increasing popularity of waterpipe tobacco smoking and electronic cigarette use: implications for oral healthcare. J Periodontal Res. (2017) 52:813–23. doi: 10.1111/jre.12458 28393367 PMC5585021

[6] Bialous SA Glantz SA. Heated tobacco products: another tobacco industry global strategy to slow progress in tobacco control. Tob Control. (2018) 27:s111–7. doi: 10.1136/tobaccocontrol-2018-054340, PMID: 30209207 PMC6202178

[7] Azzopardi D Liu C Murphy J. Chemical characterization of tobacco-free “modern” oral nicotine pouches and their position on the toxicant and risk continuums. Drug Chem Toxicol. (2022) 45:2246–54. doi: 10.1080/01480545.2021.1925691, PMID: 34034614

[8] Nebraska University Health Center. (2024). Nicotine pouches: are they safer than chewing, smoking or vaping? Available online at: https://health.unl.edu/nicotine-pouches-are-they-safer-chewing-smoking-or-vaping (Accessed June 9, 2024).

[9] Robichaud MO Seidenberg AB Byron MJ. Tobacco companies introduce “tobacco-free” nicotine pouches. Tob Control [Internet]. (2020). 29:e145–6. Available at: https://tobaccocontrol.bmj.com/content/29/e1/e145 10.1136/tobaccocontrol-2019-055321 PMC7239723 31753961

[10] Liu J Rensch J Wang J Jin X Vansickel A Edmiston J. Nicotine pharmacokinetics and subjective responses after using nicotine pouches with different nicotine levels compared to combustible cigarettes and moist smokeless tobacco in adult tobacco users. Psychopharmacology. (2022) 239:2863–73. doi: 10.1007/s00213-022-06172-y, PMID: 35869988 PMC9385814

[11] East N Bishop E Breheny D Gaca M Thorne D. A screening approach for the evaluation of tobacco-free ‘modern oral’ nicotine products using real time cell analysis. Toxicol Rep. (2021) 8:481–8. doi: 10.1016/j.toxrep.2021.02.014, PMID: 33718000 PMC7933807

[12] Gholap VV Kosmider L Golshahi L Halquist MS. Nicotine forms: why and how do they matter in nicotine delivery from electronic cigarettes? Expert Opin Drug Deliv. (2020) 17:1727–36. Available at: https://www.tandfonline.com/doi/full/10.1080/17425247.2020.1814736 32842785 10.1080/17425247.2020.1814736 PMC9361466

[13] Majmundar A Okitondo C Xue A Asare S Bandi P Nargis N. Nicotine pouch sales trends in the US by volume and nicotine concentration levels from 2019 to 2022. JAMA Netw Open. (2022) 5:e2242235. Available at: https://jamanetwork.com/journals/jamanetworkopen/fullarticle/2798449 36378312 10.1001/jamanetworkopen.2022.42235 PMC9667333

[14] Lyu JC Ozga JE Stanton CA Hrywna M Ganz O Cornacchione Ross J. Advertising the leading US nicotine pouch brand: a content analysis of ZYN advertisements from 2019 to 2023. Tob Control [Internet]. (2025). tc-2024-059145. Available at: https://tobaccocontrol.bmj.com/content/early/2025/05/06/tc-2024-059145 10.1136/tc-2024-059145 PMC12379097 40335264

[15] Stanfill S Tran H Tyx R Fernandez C Zhu W Marynak K. Characterization of Total and Unprotonated (free) nicotine content of nicotine pouch products. Nicotine Tobacco Research. (2021) 23:1590–6. doi: 10.1093/ntr/ntab030, PMID: 34233354

[16] Lawler TS Stanfill SB Zhang L Ashley DL Watson CH. Chemical characterization of domestic oral tobacco products: Total nicotine, pH, unprotonated nicotine and tobacco-specific N-nitrosamines. Food Chem Toxicol. (2013) 57:380–6. doi: 10.1016/j.fct.2013.03.011, PMID: 23517910 PMC5659123

[17] Chapman Matthew The Bureau of Investigative Journalism. (2021). New products, old tricks? Concerns big tobacco is targeting youngsters — the Bureau of Investigative Journalism (en-GB). Available online at: https://www.thebureauinvestigates.com/stories/2021-02-21/new-products-old-tricks-concerns-big-tobacco-is-targeting-youngsters (Accessed November 3, 2024).

[18] Jackler RK Chau C Getachew BD Al E. JUUL advertising over its first three years on the market: Stanford research into the impact of tobacco advertising. Stanford, CA, USA: Stanford University School of Medicine (2019).

[19] National Center for Chronic Disease Prevention and Health Promotion (US) Office on Smoking and Health. E-cigarette use among youth and young adults. Centers for Disease Control and Prevention (US) [Internet]. Available online at: www.cdc.gov/tobacco

[20] IARC Working Group on the Evaluation of Carcinogenic Risks to Humans. Smokeless tobacco and some tobacco-specific N-nitrosamines [Internet]. Vol. 89, IARC monographs on the evaluation of carcinogenic risks to humans / World Health Organization, International Agency for Research on Cancer. Lyon (FR): International Agency for Research on Cancer; (2007). 1–592. Available at: https://www.ncbi.nlm.nih.gov/books/NBK326497/PMC4781254 18335640

[21] Vishwakarma A Verma D. Microorganisms: crucial players of smokeless tobacco for several health attributes. Appl Microbiol Biotechnol. (2021). 105:6123–32. doi: 10.1007/s00253-021-11460-2, PMID: 34331556

[22] Mallock N Schulz T Malke S Dreiack N Laux P Luch A. Levels of nicotine and tobacco-specific nitrosamines in oral nicotine pouches. Tob Control. (2024) 33:193–9. Available at: https://tobaccocontrol.bmj.com/content/33/2/193 38378209 10.1136/tc-2022-057280

[23] Administration U.S. FDA. U.S. FDA. (2025). FDA authorizes marketing of 20 ZYN nicotine pouch products after extensive scientific review. Available online at: https://www.fda.gov/news-events/press-announcements/fda-authorizes-marketing-20-zyn-nicotine-pouch-products-after-extensive-scientific-review (Accessed February 16, 2025).

[24] Mallock-Ohnesorg N Rabenstein A Stoll Y Gertzen M Rieder B Malke S. Small pouches, but high nicotine doses—nicotine delivery and acute effects after use of tobacco-free nicotine pouches. Front Pharmacol. (2024) 15:1–12. doi: 10.3389/fphar.2024.1392027, PMID: 38841367 PMC11150668

[25] Morean ME Bold KW Davis DR Kong G Krishnan-Sarin S Camenga DR. Awareness, susceptibility, and use of oral nicotine pouches and comparative risk perceptions with smokeless tobacco among young adults in the United States. PLoS One. (2023) 18:1–12. doi: 10.1371/journal.pone.0281235 PMC9886243 36716297

[26] Pierce JP Choi WS Gilpin EA Farkas AJ Merritt RK. Validation of susceptibility as a predictor of which adolescents take up smoking in the United States. Health Psychol. (1996) 15:355–61. 8891714 10.1037//0278-6133.15.5.355

[27] Sparrock LS Phan L Chen-Sankey J Hacker K Ajith A Jewett B. Nicotine pouch: awareness, beliefs, use, and susceptibility among current tobacco users in the United States, 2021. Int J Environ Res Public Health. (2023) 20:2050. doi: 10.3390/ijerph20032050, PMID: 36767414 PMC9915420

[28] Digital Government Authority. The Riyadh municipality. (2025). Available online at: https://www.alriyadh.gov.sa/en/municipalities (Accessed July 15, 2025).

[29] La Rosa GRM Fagerström K Pacino SA Kowalski J Górska R Gospodaru S. Self-reported oral health outcomes after switching to a novel nicotine pouch technology: a pilot study. Acta Odontol Scand. (2025) 84:292–8. doi: 10.2340/aos.v84.43805, PMID: 40422555 PMC12138386

[30] Rusiecka AW Szymacha K Lewkowicz N. The safety of using oral nicotine pouche – consideration of their effects on general health, oral mucosa and periodontal diseases, comparing them to snus and other nicotinecontaining products. J Pre-Clin Clin Res. (2024) 18:224–30.

[31] General Authority for Statistics. (2023). Saudi Census Statistics 2022, Population Statistics. Available online at: https://www.stats.gov.sa/en/statistics-tabs?tab=436327&category=417653 (Accessed October 14, 2024).

[32] Felicione NJ Schneller LM Goniewicz ML Hyland AJ Cummings KM Bansal-Travers M. Oral nicotine product awareness and use among people who smoke and vape in the U.S. Am J Prev Med. (2022) 63:611–8. doi: 10.1016/j.amepre.2022.04.019, PMID: 35667923 PMC9509436

[33] Hrywna M Gonsalves NJ Delnevo CD Wackowski OA. Nicotine pouch product awareness, interest and ever use among US adults who smoke, 2021. Tob Control. (2023) 32:782–5. doi: 10.1136/tobaccocontrol-2021-057156, PMID: 35217596 PMC9402802

[34] Elsokkary EM Alsabhan FA Alyahya AA Alsahli SA Almousa AM Aldali JA. Exploring the effect of nicotine pouches on users’ health in Saudi Arabia: a cross-sectional study. Tob Induc Dis. (2025) 23:1–6. doi: 10.18332/tid/203510, PMID: 40352804 PMC12063099

[35] Keller-Hamilton B Alalwan MA Curran H Hinton A Long L Chrzan K. Evaluating the effects of nicotine concentration on the appeal and nicotine delivery of oral nicotine pouches among rural and Appalachian adults who smoke cigarettes: a randomized cross-over study. Addiction. (2024) 119:464–75. doi: 10.1111/add.16355, PMID: 37964431 PMC10872395

[36] Saudi Food & Drug Authority. SFDA.FD5005:2020 “E-liquids and heated tobacco in electronic nicotine delivery systems”. (2020). Available online at: https://www.sfda.gov.sa/sites/default/files/2021-10/ElectronicNicotineDeliverySystems.pdf [Accessed November 21, 2024].

[37] Vogel EA Barrington-Trimis JL Kechter A Tackett AP Liu F Sussman S. Differences in young adults’ perceptions of and willingness to use nicotine pouches by tobacco use status. Int J Environ Res Public Health. (2022) 19:2685. doi: 10.3390/ijerph19052685, PMID: 35270385 PMC8910652

[38] Rungraungrayabkul D Gaewkhiew P Vichayanrat T Shrestha B Buajeeb W. What is the impact of nicotine pouches on oral health: A systematic review, vol. 24. BioMed Central Ltd: BMC Oral Health (2024). 10.1186/s12903-024-04598-8 PMC11297755 39097712

[39] Alizadehgharib S Lehrkinder A Alshabeeb A Östberg AK Lingström P. The effect of a non-tobacco-based nicotine pouch on mucosal lesions caused by Swedish smokeless tobacco (snus). Eur J Oral Sci. (2022) 130:e12885. doi: 10.1111/eos.12885, PMID: 35853092 PMC9540014

[40] Miluna S Melderis R Briuka L Skadins I Broks R Kroica J. The correlation of Swedish snus, nicotine pouches and other tobacco products with Oral mucosal health and salivary biomarkers. Dent J. (2022) 10:154. doi: 10.3390/dj10080154, PMID: 36005252 PMC9406994

[41] Johnson GK Guthmiller JM Joly S Organ CC Dawson DV. Interleukin-1 and interleukin-8 in nicotine- and lipopolysaccharide-exposed gingival keratinocyte cultures. J Periodontal Res. (2010) 45:583–8. PMID: 20337880 10.1111/j.1600-0765.2009.01262.x

[42] Kubota M Yanagita M Mori K Hasegawa S Yamashita M Yamada S. The effects of cigarette smoke condensate and nicotine on periodontal tissue in a periodontitis model mouse. PLoS One. (2016) 11:e0155594. doi: 10.1371/journal.pone.0155594, PMID: 27203240 PMC4874667

[43] Bosco AF. A histologic and histometric assessment of the influence of nicotine on alveolar bone loss in rats. J Periodontol. (2007) 78:527–32. doi: 10.1902/jop.2007.060149, PMID: 17335377

[44] Liu J Edmiston JS Wang J Milleman KR Milleman JL Yoder AL. Oral health effects among adults switching from cigarettes to on!® nicotine pouches compared to those who continue smoking. Oral Health Prev Dent. (2025) 23:189–201. 40130808 10.3290/j.ohpd.c_1925 PMC11966149

[45] Edmiston J Liu J Wang J Sarkar M. A randomized, controlled study to assess biomarkers of exposure in adult smokers switching to Oral nicotine products. J Clin Pharmacol. (2022) 62:1445–58. doi: 10.1002/jcph.2098, PMID: 35730535 PMC9804531

[46] PROTEX® S. Stingfreesnus.Com. (2025). Stingfree Strong Blue Mint. Available online at: https://stingfreesnus.com/strong-blue-mint/ (Accessed July 15, 2025).

[47] KLAR®. (2025). KLAR® Bioceramic Nicotine Pouches. Available online at: https://www.klar.ing/why-bioceramic (Accessed July 14, 2025).

[48] Franzen A Loof J Birgersson U Kiselovs G Pokosta M Engqvist H. Comparative study of calcium sulphate and cellulose based nicotine pouch on pharmacokinetics, pulse rate and nicotine extraction. Discover Medicine [Internet]. (2025). 2:193. Available online at: https://www.researchsquare.com/article/rs-5953198/v1

