# Supplementary material for: Investigating oral nicotine pouch use among adults in Riyadh, Saudi Arabia: prevalence, awareness, susceptibility, and associated symptoms
Source: Front Public Health. 2025 Sep 4;13:1607656. doi: 10.3389/fpubh.2025.1607656 (PMC12445106; doi:10.3389/fpubh.2025.1607656)
Supplement: Supplementary file 1 [file Table_1.docx]

**Nicotine Pouch Questionnaire: Awareness, Use Patterns, and Perceptions among Saudi Adults**

Simplified table describing ONP survey items:

| **Survey section** | **Item description** | **Response options** | **Classification criteria** |
| --- | --- | --- | --- |
| **ONP awareness** | Seen or heard of nicotine pouches before the study (with visual aid of popular brands) | Yes / No / Not Sure | "Yes" → Aware.  "No"/"Not Sure" → Unaware. |
| **Susceptibility (non-users only)** | 1. Interest in trying ONPs 2. Perceived information sufficiency about ONP risks 3. Willingness to use if offered | Yes / No for each item | *Non-susceptible* if they indicated no interest, reported sufficient knowledge of ONP health risks, and stated an un-willingness to use;  All others were classified as *susceptible*. |
| **Beliefs about ONPs** | 1. Perceived health risks 2. Perceived value as cessation aid 3. Perceived addiction potential | 5-point Likert scale: Strongly agree, agree, don’t know, strongly disagree and disagree. | Responses grouped as: - Agree (combining “strongly agree” and “agree”).  - Disagree (combining “strongly disagree” and “disagree”)  - Don’t know”. |
| **ONP user-reported symptoms** | Symptoms from ONP use (self-report) | - Frequent coughing - Oral irritation - Taste changes - Dry mouth - Throat symptoms - Abdominal symptoms - No symptoms - Other symptoms | All reported symptoms listed with options for “No symptoms” and “Other”. |

**Questions:**

(Note: the informed consent form was provided before starting the questionnaire that clearly stating the study's purpose, confidentiality, voluntary participation, participant rights and contact information)

**Section 1: Demographics and Screening**

1. Gender:

- Male
- Female

1. Age Group:
   - 18-29 years
   - 30-39 years
   - 40-69 years
2. Please specify the name of district you currently reside in within the Riyadh city (e.g., Olaya, Namar, Annasim District):

-------------------------------------------------------------------------------------------------

1. Smoking Status: Which of the following tobacco or nicotine products do you currently use or have ever used? (Select all that apply)

- Cigarettes (smoking)
- Electronic cigarettes (vapes/e-cigarettes)
- Shisha (Hookah/Mu'assel)
- I do not currently use any tobacco or nicotine products.

**Section 2.1: Awareness of Nicotine Pouches**

1. Have you ever heard of nicotine pouches? (An image displaying examples of popular nicotine pouch brands was provided here to ensure clarity)

- Yes
- No

1. Do you know anyone in your social circle (friends, family, colleagues) who uses nicotine pouches?

- Yes
- No
- Unsure

**Section 2.2: Nicotine Pouch Use Patterns**

1. Have you ever used nicotine pouches?

- Yes
- No

1. How many nicotine pouches do you use per day? (Please answer if you are a nicotine pouch user)

- 1 pouch
- 2 pouches
- 3 pouches
- 4 pouches
- 5 and More pouches

1. If you are a nicotine pouch user, have you experienced any of the following symptoms while using nicotine pouches? (Select all that apply)

- Frequent coughing
- Oral irritation (eg. white patches in the mouth, gum irritation/recession, blisters)
- Change in sense of taste
- Dry mouth
- Throat irritation/soreness
- Abdominal discomfort (eg. stomach/abdominal pain or digestive issues)
- Other symptoms (please specify:……….)
- I have not experienced any symptoms
- Prefer not to answer

**Section 2.3: Perceived Health Risks and Beliefs**

1. Nicotine pouches pose a health risk.

- Strongly Disagree
- Disagree
- Don't know
- Agree
- Strongly Agree

1. Nicotine pouches are more harmful than traditional cigarettes and shisha. *(Scale 2: only provided for the pilot study)*

- Strongly Disagree
- Disagree
- Don't know
- Agree
- Strongly Agree

1. Nicotine pouches are addictive.

- Strongly Disagree
- Disagree
- Don't know
- Agree
- Strongly Agree

1. I believe one can easily become dependent on nicotine pouches. *(Scale 2: only provided for the pilot study)*

- Strongly Disagree
- Disagree
- Don't know
- Agree
- Strongly Agree

1. Nicotine pouches can help people quit smoking traditional cigarettes.

- Strongly Disagree
- Disagree
- Don't know
- Agree
- Strongly Agree

1. Using nicotine pouches is an effective way to reduce cigarette consumption. *(Scale 2: only provided for the pilot study)*

- Strongly Disagree
- Disagree
- Don't know
- Agree
- Strongly Agree

**Section 2.4: Information & Future Interest**

1. Do you feel you have sufficient information about the potential risks of nicotine pouches?

- Yes
- No
- Unsure

1. Are you interested in trying nicotine pouches?

- Yes
- No

1. Would you consider using nicotine pouches if offered by a friend or readily available?

- Yes
- No

1. Would you like to get more information about nicotine pouches?

- Yes
- No

1. To help us ensure the accuracy of participant data, please select the correct answer: What does 2 + 2 = ?

- 5
- 8
- 10
- 4
